# The resurgence of the Adora2b receptor as an immunotherapeutic target in pancreatic cancer

**DOI:** 10.3389/fimmu.2023.1163585

**Published:** 2023-04-28

**Authors:** Lincoln N. Strickland, Erika Y. Faraoni, Wei Ruan, Xiaoyi Yuan, Holger K. Eltzschig, Jennifer M. Bailey-Lundberg

**Affiliations:** Department of Anesthesiology, Critical Care, and Pain Medicine, McGovern Medical School, The University of Texas Health Science Center at Houston, Houston, TX, United States

**Keywords:** immunotherapy, pancreatic adenocarcinoma, hypoxia, Adenosine receptor 2B, CD8+ T cell response

## Abstract

Pancreatic ductal adenocarcinoma (PDAC) is characterized by a dense desmoplastic stroma that impedes drug delivery, reduces parenchymal blood flow, and suppresses the anti-tumor immune response. The extracellular matrix and abundance of stromal cells result in severe hypoxia within the tumor microenvironment (TME), and emerging publications evaluating PDAC tumorigenesis have shown the adenosine signaling pathway promotes an immunosuppressive TME and contributes to the overall low survival rate. Hypoxia increases many elements of the adenosine signaling pathway, resulting in higher adenosine levels in the TME, further contributing to immune suppression. Extracellular adenosine signals through 4 adenosine receptors (Adora1, Adora2a, Adora2b, Adora3). Of the 4 receptors, Adora2b has the lowest affinity for adenosine and thus, has important consequences when stimulated by adenosine binding in the hypoxic TME. We and others have shown that Adora2b is present in normal pancreas tissue, and in injured or diseased pancreatic tissue, Adora2b levels are significantly elevated. The Adora2b receptor is present on many immune cells, including macrophages, dendritic cells, natural killer cells, natural killer T cells, γδ T cells, B cells, T cells, CD4^+^ T cells, and CD8^+^ T cells. In these immune cell types, adenosine signaling through Adora2b can reduce the adaptive anti-tumor response, augmenting immune suppression, or may contribute to transformation and changes in fibrosis, perineural invasion, or the vasculature by binding the Adora2b receptor on neoplastic epithelial cells, cancer-associated fibroblasts, blood vessels, lymphatic vessels, and nerves. In this review, we discuss the mechanistic consequences of Adora2b activation on cell types in the tumor microenvironment. As the cell-autonomous role of adenosine signaling through Adora2b has not been comprehensively studied in pancreatic cancer cells, we will also discuss published data from other malignancies to infer emerging therapeutic considerations for targeting the Adora2b adenosine receptor to reduce the proliferative, invasive, and metastatic potential of PDAC cells.

## Introduction

Pancreatic ductal adenocarcinoma (PDAC) is a lethal malignancy, with only a 3-13% 5-year survival rate, which is critically dependent on the stage at diagnosis. PDAC is characterized by a highly immunosuppressive and hypoxic tumor microenvironment. Risk factors include age, chronic pancreatitis, diabetes, genetic predisposition, obesity, and smoking (1, 2). Current therapeutic approaches including chemotherapy and radiation have not resulted in significant changes in overall survival, highlighting the continued need for testing new therapeutic strategies to treat PDAC patients. In this review, we will expand on an immune suppressive pathway in PDAC, the adenosine signaling pathway, with a focus on the role of the Adora2b receptor. Work from our lab and others has shown this pathway is elevated in a subset of patients with PDAC, and inhibition of extracellular adenosine generation augments anti-tumor immunity in several preclinical pancreatic cancer models (3–6). We will discuss the mechanistic consequences of elevated extracellular adenosine in the pancreatic cancer microenvironment and will emphasize emerging considerations for targeting the Adora2b receptor as a therapeutic target to improve outcomes for patients at high risk or who have been diagnosed with PDAC (7–9).

**Figure 1:**
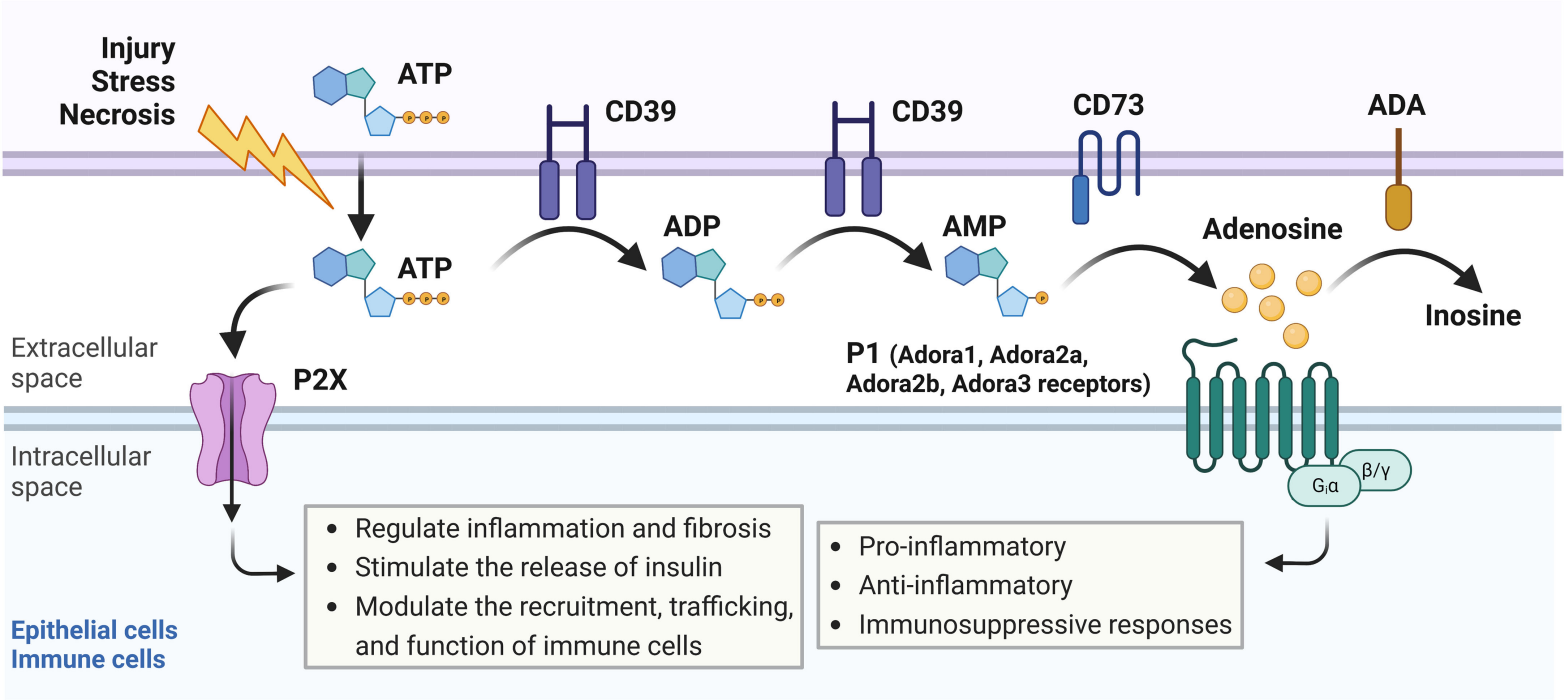
Adenosine signaling pathway overview. In response to cellular injury, stress, or necrosis, adenosine triphosphate (ATP) is released to the extracellular space, where it can signal through P2X receptors on epithelial or immune cells to regulate inflammation and fibrosis, stimulate the release of insulin, or modulate recruitment, trafficking, and function of immune cells. Extracellular ATP can also be converted by CD39, an ectonucleotidase enzyme, into adenosine diphosphate (ADP) or adenosine monophosphate (AMP). AMP can then be converted into adenosine by CD73, another ectonucleotidase enzyme. Adenosine binds to P1 receptors including Adora1, Adora3, Adora2a, or Adora2b, which can all be expressed on epithelial, stromal, or immune cells. Activation of the P1 receptors results in pro-inflammatory, anti-inflammatory, or immunosuppressive responses depending on which P1 receptor is involved and which intracellular signaling pathways are activated downstream. Adenosine can also be converted to inosine by adenosine deaminase (ADA) in an irreversible termination process.

Heterocyclic aromatic molecules such as adenosine triphosphate (ATP), adenosine diphosphate (ADP), and adenosine are purines essential to life, indispensable for maintaining intracellular energy balance, cellular processes, and pathways (10). ATP is generated by glycolysis or oxidative phosphorylation and is commonly known as the principal molecule for storing and transferring energy in the cell (11). Within the cell, ATP molecules are transported by mitochondrial ADP/ATP carriers (AAC) proteins, major components of the inner mitochondrial membrane that regulate ATP synthesis by influencing ADP intake in the mitochondria. In the contexts of cellular injury, stress, hypoxia, or cell death, ATP can be secreted out of the cell in exosomes (exocytotic release), through connexin or pannexin channels, or by volume-regulated anion channels to the extracellular space, where it signals through purinergic receptors and participates in a broad range of cellular processes (12, 13). Some of the roles of extracellular ATP include the regulation of inflammation and fibrosis (14). Both ATP and extracellular ADP can be converted by an ectonucleotidase enzyme (CD39) into adenosine monophosphate (AMP), a molecule that can then be converted to adenosine by ecto-5’-nucleotidase (CD73) (**Figure 1**) (11, 15). Adenosine has been shown to participate in pro-inflammatory, anti-inflammatory, fibrotic, and immunosuppressive responses dependent on cell type activated, extracellular concentrations of ATP, ADP, and adenosine, degree of hypoxia, and availability and duration of binding to P1 receptors including Adora1, Adora3, Adora2a or Adora2b which can all be expressed on epithelial, stromal, or immune cells. Such responses vary depending on the P1 receptor involvement and intracellular signaling downstream of receptor activation (15–18). Extracellular adenosine signaling can be terminated through the uptake of adenosine into cells through two predominant equilibrative nucleoside transporters (ENTs), ENT1 and ENT2, which are bidirectional transport channels that allow transmembrane diffusion of nucleosides (19, 20). Termination of adenosine signaling can also occur when adenosine undergoes an irreversible termination process by the enzyme adenosine deaminase (ADA), which converts adenosine to inosine (21).

## Hypoxia-mediated adenosine signaling in inflammatory and tumor microenvironments

Hypoxia is a hallmark of chronic inflammatory conditions including several solid tumors; yet hypoxic conditions can occur in the early stages of inflammation due to the oxygen requirements of neutrophils and other immune cells, causing nearby epithelial and stromal cells to become oxygen-depleted (22). Chronic inflammation exacerbates this response resulting in hypoxia-inducible factor (HIF) activation in immune, stromal, and epithelial cells. Hypoxia-inducible factor 1-alpha (HIF-1α) is a well-known regulator of hypoxic cellular processes, and its activity is mainly controlled by post-translational rather than transcriptomic modifications. During normoxic conditions, HIF-1α levels are kept low by the Von Hippel-Lindau (VHL) tumor suppressor which targets HIF-1α for ubiquitin-mediated proteasomal degradation. However, when oxygen levels become depleted, HIF-1α starts to accumulate and HIF-1α stabilizes and binds to HIF-1β forming a complex that enters the nucleus and binds to hypoxia response elements (HRE) to either promote or repress genes (23, 24). In a mouse model of caerulean-induced acute pancreatitis, injured tissues presented high expression of HIF-1α, and inhibition of HIF-1α, through intraperitoneal injections of HIF-1α small molecule inhibitor PX478, reduced RIP3/p-MLKL expression and ROS production, mitigating acinar cell injury and necrosis (25). In the context of pancreatic cancer, HIF-1α levels are elevated in part due to the desmoplastic stroma and HIF-1α staining and expression strongly associates with PDAC lymph node metastasis, high tumor stage, poor prognosis, and immune evasion (26). A recent study in an autochthonous mouse model of PDAC with pancreas-specific expression of *Kras* ^G12D^ implicates HIF-1α may have a protective role, as genetic deletion of the gene promotes neoplasia. Immunohistochemical staining and ELISA analysis revealed that HIF-1α genetic deletion significantly increases secretion of the B-cell chemoattractant CXCL13, which increases the intrapancreatic accumulation of B cells, as shown through flow cytometry analysis. These data indicate HIF-1α prevents B cell infiltration into hypoxic regions and when B cells were depleted in mice, PanIN development was decreased, implicating B cells promote tumorigenesis in PDAC (27). The expression of Adora2b and its subsequent activation was shown to be elevated by HIF-1α in hepatic ischemia-reperfusion injury mouse models, acute lung injury, liver cancer, and breast cancer (28). During pancreatic diseases, hypoxic conditions tend to develop and both HIF-1α and Adora2b are elevated and involved in the inflammatory process (4, 29), yet, further analysis is needed to fully uncover the potential link between both molecules and their participation in the development of these diseases.

**Figure 2:**
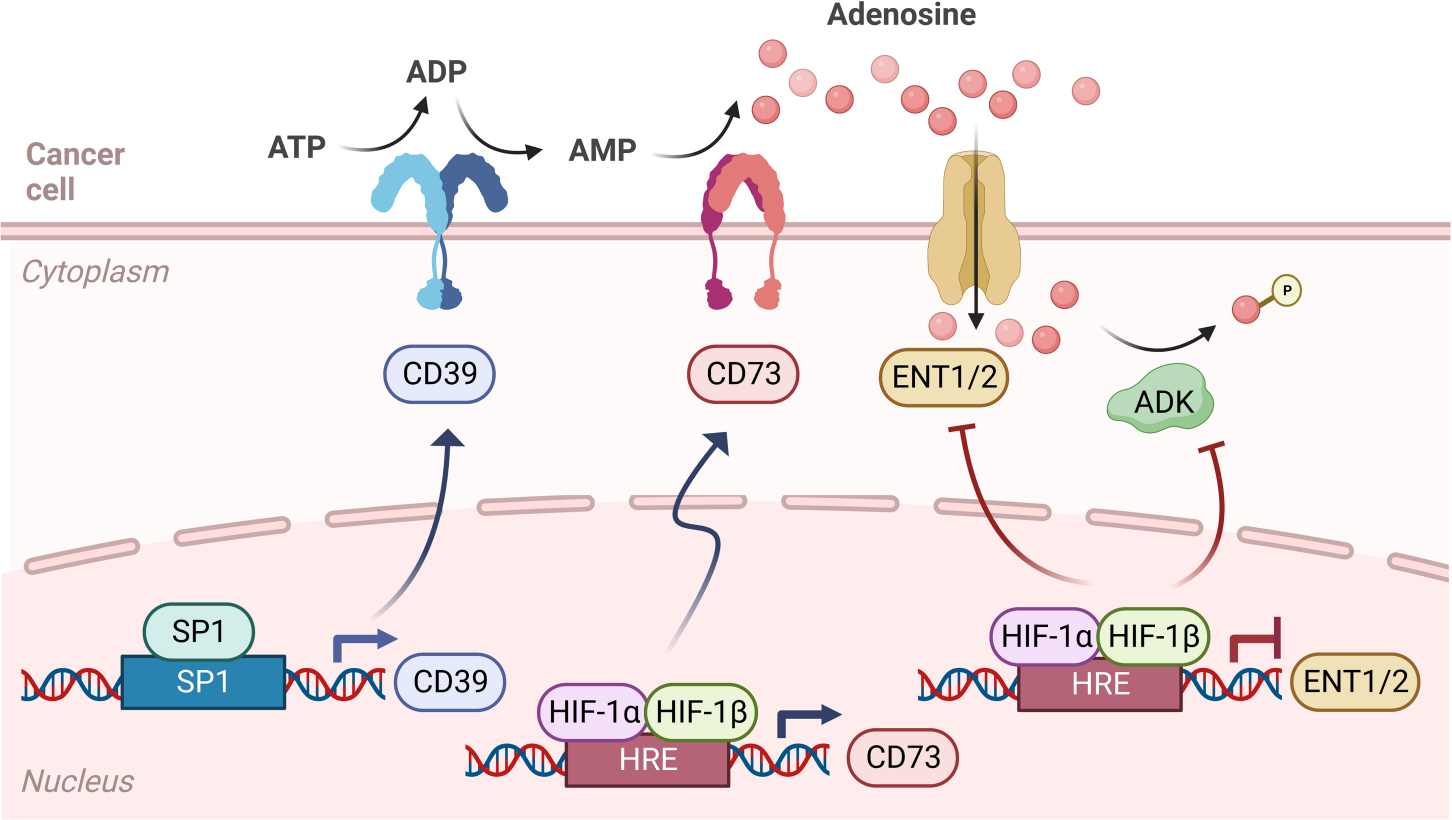
Adenosine signaling pathway during hypoxia. Adenosine signaling in hypoxia is similar to normoxia, as ATP is converted to ADP and AMP by CD39, then converted to adenosine by CD73. However, in hypoxic cancer cells, the transcription of CD39 is upregulated through Sp1, leading to more ADP and AMP in the tumor microenvironment (TME). Also, while levels of HIF-1α are kept low by the Von Hippel-Lindau (VHL) tumor suppressor in normoxic conditions, in hypoxia HIF-1α stabilizes and binds to HIF-1β, which forms a complex that enters the nucleus and binds to hypoxia response elements (HRE) on the gene promoter, therefore regulating the transcription of CD73 and equilibrative nucleoside transporters (ENT1/2). In hypoxia, CD73 transcription is upregulated, while ENT1 and ENT2 transcription is downregulated. HIF-1α also inhibits adenosine kinase and ENTs, leading to an accumulation of adenosine in the TME.

Studies of hypoxia-induced changes in gene expression identified a transcriptional program that promotes CD73 expression in the extracellular vicinity of inflamed tissues (**Figure 2**). In these studies, Adora2b gene expression is also elevated resulting in an endogenous feedback loop critical for injury resolution and ischemia tolerance under oxygen-deprived conditions (30–32). Transcription of CD73 is regulated by an HRE on the promoter in hypoxic epithelial cells and transcription of CD39 is either upregulated through Sp1 or downregulated through the formation of a HIF-1α and AHR complex with ARNT which decreases AHR recruitment to the CD39 promoter that has three AHR response elements (33–35). HIF-1α inhibits adenosine kinase and ENTs resulting in increased accumulation of adenosine in the tumor microenvironment (19, 20, 36). Another ligand for Adora2b is Netrin-1, a neuronal guidance molecule essential for the proper development of neurons. In PDAC, perineural infiltration is present in early and late stages of the disease and neuronal infiltration by tumor cells may contribute to pain and tumor progression indicating Netrin-1/Adora2b signaling could be evaluated as a therapeutic strategy to reduce perineural infiltration. In addition, signaling of Netrin-1 through the Adora2b receptor also inhibits immune cell infiltration into organs under hypoxic and inflammatory conditions (37–39) indicating several mechanistic consequences for Adora2b in pancreatic and other solid tumors. In addition to Netrin, *in vitro* data have shown that stimulation and activation of Adora2b by adenosine and NECA promotes cell proliferation and secretion of chromogranin A, a protein that is widely accepted as a biomarker for neuroendocrine tumors. Such findings suggest inhibition of the adenosine pathway, specifically targeting Adora2b receptors, may be of high interest in the therapeutic management of neuroendocrine tumors (40).

Another component of the PDAC TME is the vasculature, which is characterized by high microvascular density yet poor perfusing in the vessels and decreased vascular integrity. In PDAC patients, the superior mesenteric vessels are commonly involved, especially when tumors arise in the head of the pancreas. These clinical features of PDAC are notable in the context of adenosine signaling as hypoxia-mediated adenosine signaling influences vascular responses. In the context of inflammation, neutrophils exit the bloodstream through transendothelial migration (TEM) and secrete ATP and ADP resulting in high adenosine concentrations (41–44). Studies exploring the role of adenosine receptors in vascular leakage were completed in mice that were deficient in either Adora1, Adora3, Adora2a, or Adora2b, then subjected to hypoxia. While the Adora1, Adora3, or Adora2a mice did not have an increase in hypoxia-induced vascular leakage, the Adora2b deficient mice showed a significant increase in hypoxia-induced vascular leakage. Furthermore, administration of the Adora2b antagonist PSB1115 to wild-type mice also significantly increased neutrophil infiltration through TEM and worsened vascular leakage while administration of Adora2b agonist BAY-60-6583 reversed the hypoxia-induced vascular leakage. These findings suggest Adora2b has a key role in controlling hypoxia-associated vascular leak by increasing endothelial cell intracellular levels of cAMP which promotes vasculature resealing (31, 45). These studies suggest adenosine signaling events can be targeted to dampen hypoxia-induced inflammation and prevent excessive tissue damage (13, 30). In solid tumors with a hypoxic TME, Adora2b antagonists may promote increased infiltration of immune cells and anti-tumor immunity.

## Functional consequences of adenosine receptor signaling in inflammation and cancer

### Adora1 and Adora3 receptors

The Adora1, or adenosine A_1_ receptor, is a G protein-coupled receptor (GPCR) that, when bound to an agonist, causes G_i1,2,3_ or G_0_ protein binding. Adora1 is ubiquitously expressed in the body and, when G_i1,2,3_ is bound, adenylate cyclase is inhibited, and cAMP concentrations are decreased. This has important consequences in several fundamental biological contexts including slowing heart rate (46, 47), reducing glucose-induced insulin secretion (48), reducing blood flow, and promoting edema during acute pancreatitis (49). In the context of cancer, Adora1 overexpression has been published to facilitate the malignant progression of colorectal, kidney, and breast cancers, as well as glioblastoma and leukemia (50). Inhibition of Adora1 in combination with immune checkpoint blockade (ICB) therapy targeting PD-1 has shown promising therapeutic effects in non-small cell lung cancer and melanoma (51). In contrast, studies evaluating the role of hypoxia in the pancreas reveal Adora1 is downregulated during hypoxia (52) and analysis of RNA-seq data from The Cancer Genome Atlas (TCGA) database indicated this receptor was not associated with PDAC prognosis (48). Thus, the role of Adora1 in response to hypoxia or other environmental triggers of adenosine is dependent on tumor type and organ of origin.

The Adora3 or adenosine A_3_ receptor couples to Gi/Gq proteins. Like Adora1, Adora3 receptor activation promotes Gi protein binding and decreased adenylyl cyclase activity which reduces cAMP intracellular levels. Adenosine signaling through Adora3 has been shown to participate in the degranulation and activation of mast cells important in asthma pathogenesis (53–55). Adora3 also modulates cytokine release *via* T cell-mediated production of IL-10 which helps reverse neuropathic pain (56) and through down-regulation of nuclear factor-kappa B signaling results in the inhibition of inflammatory cytokine production in the colonic mucosa of patients with ulcerative colitis (57). Unlike the Adora1 receptor, hypoxic conditions do not affect Adora3 expression (52). In the context of the pancreas, low levels of Adora3 receptor expression have been reported and Adora3 is not associated with PDAC prognosis (48).

### Adora2 receptors

Adora2 adenosine receptors consist of the adenosine A2_A_ (Adora2a) and A2_B_ (Adora2b) receptors, both of which are Gs-coupled GPCRs. In the pancreas, Adora2a and Adora2b have many similarities, as they both are present in the luminal membrane of ductal, insulin-positive beta, and PECAM-+ endothelial cells (11). Agonist binding to Adora2 receptors stimulates cAMP, a membrane-associated protein kinase A (type II PKA), and cAMP-activated Cl^-^ channels which mediate critical pancreatic ductal secretions (48). Adora2a is the most abundant adenosine receptor in the pancreas and it participates in endocrine pancreatic functions as well as water and bicarbonate secretion responses (48). Adora2a is also a potent anti-inflammatory regulator as its activation limits immune cell activity during an inflammatory response preventing additional tissue damage (16, 58, 59). In studies carried out in mice lacking Adora2a receptors, behavioral alterations are present, suggesting the participation of Adora2a in regulating neuronal populations (60). In caerulein-mediated mouse models of pancreatitis, inhibition of adenosine uptake using a pharmacologic inhibitor enhanced stimulation of the Adora2a receptor, and was capable of reducing the severity of pancreatitis (61). Specifically, in pancreatic cancer patients, studies show CD73 and Adora2a expression on neoplastic or tumor cells correlates with divergent immune cell populations in the tumor microenvironment. In a publication by Sweed et al, when Adora2a is overexpressed in human PDAC patients, there are correlative high levels of tumor-infiltrating mononuclear cells (TIMC), associated with larger tumor sizes (62). Moreover, in an immunohistochemical study performed on 48 human PDAC tissues, Adora2a was overexpressed, and high Adora2a PDAC expression was associated with more aggressive cases and later tumor stages at the time of diagnosis (62). While no functional experiments were reported in this manuscript, these data indicate both autocrine and paracrine adenosine signaling through Adora2a are important in the pathogenesis of pancreatic cancer.

The Adora2b receptor is the only low-affinity adenosine receptor [Adora2b EC_50_ = 24 µM, Adora2a EC_50_ = 0.7 µM, Adora1 EC_50_ = 0.31 µM, Adora3 EC_50_ = 0.29 µM (63)], requiring high levels of extracellular adenosine to become activated rather than existing in a resting state (64). Adora2b is present in myocardial cells, epithelial cells, fibroblasts, and several immune cell types (65) and in many disease models is a potent anti-inflammatory regulator. However, controversial findings exist around its role in disease, fibrosis, and tumor development. Across several mouse models of acute injury, Adora2b activation has shown protective effects, either by modulating IL-10 production on the intestinal epithelium (66), stabilization of circadian rhythm protein (67) or enhancing alveolar fluid clearance in mice (68). Additionally, studies in Adora2b deficient mice showed enhanced pulmonary recruitment of effector T cells and failed induction of regulatory T cells during endotoxin-induced inflammation resulting in increased severity of the disease. Similarly, in a pulmonary disease mouse model, induction of Adora2b signaling attenuated inflammation and edema only in wild-type mice but not in mice lacking expression of the receptor (69, 70). Contrarily, the absence of Adora2b in an ulcerative colitis mouse model ameliorated acute intestinal inflammation, suggesting this receptor plays a pro-inflammatory role in the development of this disease (71, 72).

In cancer, there are also conflicting studies related to the function of Adora2b in the progression of different malignant diseases. High Adora2b levels are associated with a better prognosis in patients with ovarian cancer. *In vitro* pharmacological activation of Adora2b in ovarian carcinoma cells reduced cell migration and actin stress fiber expression (7). However, detrimental effects were observed for mammary carcinoma, hepatocellular carcinoma, lung adenocarcinoma (LUAD), and PDAC. Adenosine signaling through Adora2b in breast cancer cells regulates the tumor microenvironment and enhances pro-tumorigenic actions in cancer-associated fibroblasts, effects correlated with increased metastatic potential and poor prognosis (73). In hepatocellular carcinoma, Adora2b receptor blockage enhanced the benefits of sorafenib treatment by suppressing the inhibitory effects of adenosine on CD8^+^ T cells (74). Bioinformatic studies in LUAD and PDAC revealed Adora2b expression and associated signaling pathways predicted poor prognosis and significantly reduced overall survival (48, 75).

## The function of Adora2b receptor on immune cells: implications for targeting to promote anti-cancer immunity

### Adora2b in innate immunity

**Figure 3:**
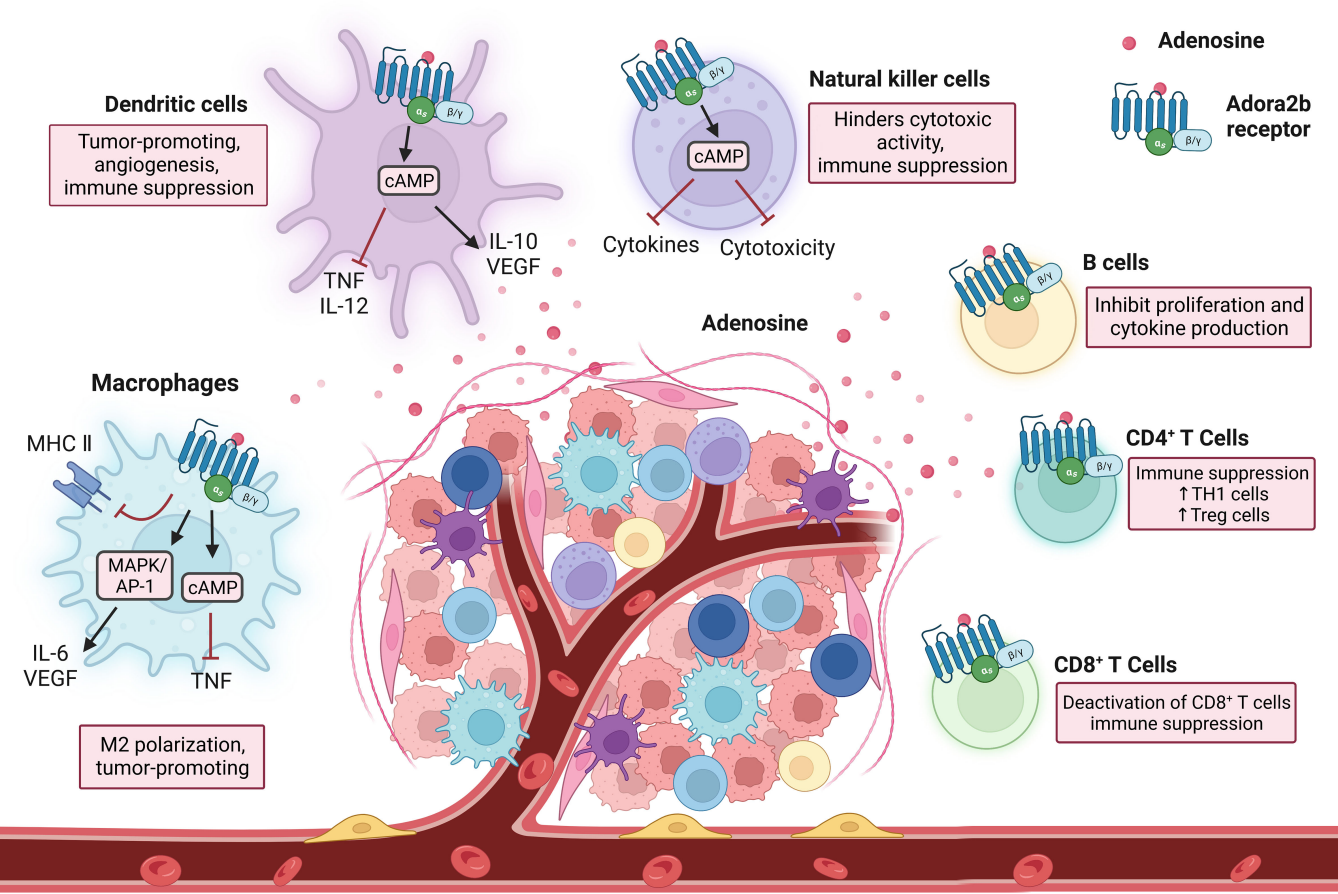
Immune cell interactions in response to Adora2b activation. As immune cells enter into the TME and encounter adenosine through the Adora2b receptor, they undergo changes resulting in immunosuppression. Adora2b activation on macrophages contributes to M2 polarization and tumorigenesis, as it inhibits MHC II expression, activates MAPK/AP-1 which increases IL-6 and vascular endothelial growth factor (VEGF) production, and increases cAMP levels which inhibits tumor necrosis factor (TNF) production. On dendritic cells, stimulation of Adora2b increases cAMP production which inhibits TNF and IL-12 production and increases IL-10 and VEGF release, resulting in tumorigenesis, angiogenesis, and immune suppression. Natural killer cells encounter adenosine through the Adora2b receptor and the cAMP pathway is activated resulting in blocked cytokine production and cytotoxicity, contributing to immunosuppression. On B cells, Adora2b activation results in the inhibition of proliferation and cytokine production. CD4^+^ T cell activation of Adora2b increases TH1 cell and Treg cell levels, as well as contributes to immune suppression. On CD8^+^ T cells, Adora2b activation results in the deactivation of CD8+ T cells and contributes to the suppression of the immune system.

Comprised of many cell types including macrophages, dendritic cells, natural killer cells, natural killer T cells, γδ T cells, and more, the innate immune system provides a rapid response to foreign antigens, and the innate immunity antitumor response triggers effector mechanisms to contain the tumor. Adenosine binding to the Adora2b receptor has important functional consequences on innate immune cells (**Figure 3**). Macrophages impart critical functions in the resolution of inflammation and a return to normal tissue conditions. Their principal function is to clear dead cells from inflamed tissues through a process called efferocytosis, which also resolves inflammation by suppressing pro-inflammatory cytokines and stimulating anti-inflammatory cytokines. Adora2b on bone marrow-derived macrophages (BMDM) was discovered through flow cytometry experiments done in mice and functionality of the receptor was assessed by Adora2b agonist, 5’-N-ethylcarboxamidoadenosine (NECA), which resulted in increased cAMP levels in cultured BMDM (76, 77). Adora2b is upregulated on macrophages by IFN-γ and when Adora2b is activated, TNF production in infiltrating macrophages is suppressed, inhibiting their capacity to secrete cytokines important for anti-tumor immunity and promoting tumor growth (78).

Dendritic cells (DCs) are antigen-presenting cells and critical determinants of both innate and adaptive immunity. They dwell in peripheral tissues in an immature state and, when exposed to triggers, transform into differentiated and mature DCs. Stimulation of Adora2b on DCs stimulates maturation into a differentiated population with DC markers and monocyte or macrophage markers, allowing mature DCs to interact with T lymphocytes and promote CD4+ differentiation into Th1 cells through IL-12 production. DCs differentiated due to exposure to adenosine have decreased allostimulatory activity and express high levels of angiogenic, immune suppression, pro-inflammatory, and tolerogenic factors, such as COX-2, IDO, IL-6, IL-8, IL-10, TGF-β, and VEGF (79, 80).

Natural killer (NK) cells are critical in responses to stress and infections. Many types of NK cells have NK receptors (NKRs) that determine if a cell encountered by an NK cell becomes a target for destruction or is protected (81). When activated NK cells encounter adenosine through the Adora2b receptor, the cAMP pathway is activated and cytotoxic activity and cytokine production is blocked, contributing to reduced anti-tumor activity (82, 83). While NKs and natural killer T cells (NKTs) have many similarities, they are very different in the context of cancer. Both cell types display effector properties in early cancer stages and have impaired functionality in later stages. NKT cells become exhausted in advanced cancers and have an irregular metabolism. NKTs have exhaustion markers such as high CTLA4, PD1, and Tim3, as well as low granzyme B levels, and reduced cell numbers as cancer progresses further (84). Limited studies have been done assessing the role of the Adora2b receptor in NKT cells.

γδ T cells are a rare subtype of T cells, bridging the gap between the innate and adaptive immune system components, they possess both γ and δ T cell receptor chains. They have gained traction in the area of immunotherapy as they have an anti-tumor immune function and are critical in immune surveillance. Analysis of TCGA data has shown PDAC patients with high CD73 levels have lower amounts of γδ T cells (85). These cells are regulated by extracellular adenosine levels, and in mice treated with an Adora2b agonist, the DCs activate γδ T cells, elevating Th17 responses (86). When γδ T cells induce an elevated Th17 response, this contributes to the pathogenesis of autoimmune diseases and can be a target in inflammation-related diseases such as cancer. However, the specific role of the Adora2b receptor in this cell type is unknown and should be explored further.

### Adora2b in adaptive immunity

Comprised of B cell and T cell subtypes, the adaptive immune system is responsible for recognizing and attacking specific antigens. B cells are lymphocytes that produce antibodies tagging specific antigens for destruction and play an important role in hypoxia and inflammation in the TME in PDAC. B cells express both CD73 and CD39, and the production of extracellular adenosine by B cells can inhibit T cell proliferation and the production of IL-10 cytokines. However, B cells have very low levels of the Adora2b receptor and few studies have been conducted to determine its role in B cell interactions (87).

T cells are a crucial group of cells in the immune system that generally express CD73, CD39, and the Adora2b receptor. The presence of Adora2b on T cells was confirmed through flow cytometry and the functionality of the receptor was determined by increased cAMP levels in the cells induced by an Adora2b agonist. Extracellular adenosine limits T cell mobility and increases cAMP levels in T cells, contributing to Adora2b-mediated immune suppression (87, 88). Helper T cells are CD4^+^ T lymphocytes that stimulate other immune cells to respond to infection and when activated, Adora2b receptor levels increase on the CD4^+^ T cell surface (88). In a model of endotoxin-induced pulmonary inflammation, mice with a genetic knockout of Adora2b had an enhanced CD4^+^ T cell response, resulting in increased inflammation (69). Adora2b on CD4^+^ T cells contributes to immunosuppression and could be a target in cancer, but additional studies are needed to learn more about the role of the receptor on CD4^+^ T cells. Cytotoxic T cells are CD8^+^ T cells that are important in protection against tumor growth, as they trigger apoptosis of pathogenic cells. In an *in vitro* experiment, activation of CD8^+^ T cells through an unspecific activation signal (phytohemagglutinin) and by a specific activation signal (the anti-T cell receptor/CD3 complex mAb, OKT3) triggers increased Adora2b levels and a decrease in IL-2 production (88). Through TCGA and The Cancer Immune Atlas analyses, PDAC patients with high CD73 levels had lower amounts of CD8^+^ T cells (4, 85). In studies performed in mice with genetic deletion of *Adora2b*, when murine PDAC cell lines derived from *Pdx1:Cre; LsL-Kras^G12D^;LsL-Trp53^R172H/+^* (KPC) mice, were implanted subcutaneously, tumor growth was significantly reduced compared to implanted cells in WT mice and there was a significant increase in Granzyme B (GZM+) and CD8^+^ T cells in KPC-derived tumors implanted in *Adora2b^-/-^* mice (4). These data indicate paracrine adenosine Adora2b signaling restrains cytotoxic CD8^+^ T cell function. Also, in complimentary studies, wild-type mice treated with PSB1115, an Adora2b antagonist, had reduced KPC subcutaneous tumor growth compared to vehicle-treated KPC tumor-bearing mice. However, in wild-type mice without CD8^+^ T cells, treatment with the PSB1115 did not inhibit the growth of the KPC subcutaneous tumors indicating paracrine adenosine signaling through Adora2b on CD8^+^ T cells reduces their anti-tumor properties in PDAC (4). Future studies using genetic models or orthotopic implantation of KPC cells into the pancreas will aid in further delineating the role of Adora2b in pancreatic cancer.

## Adora2b function in exocrine pancreatic diseases

**Figure 4:**
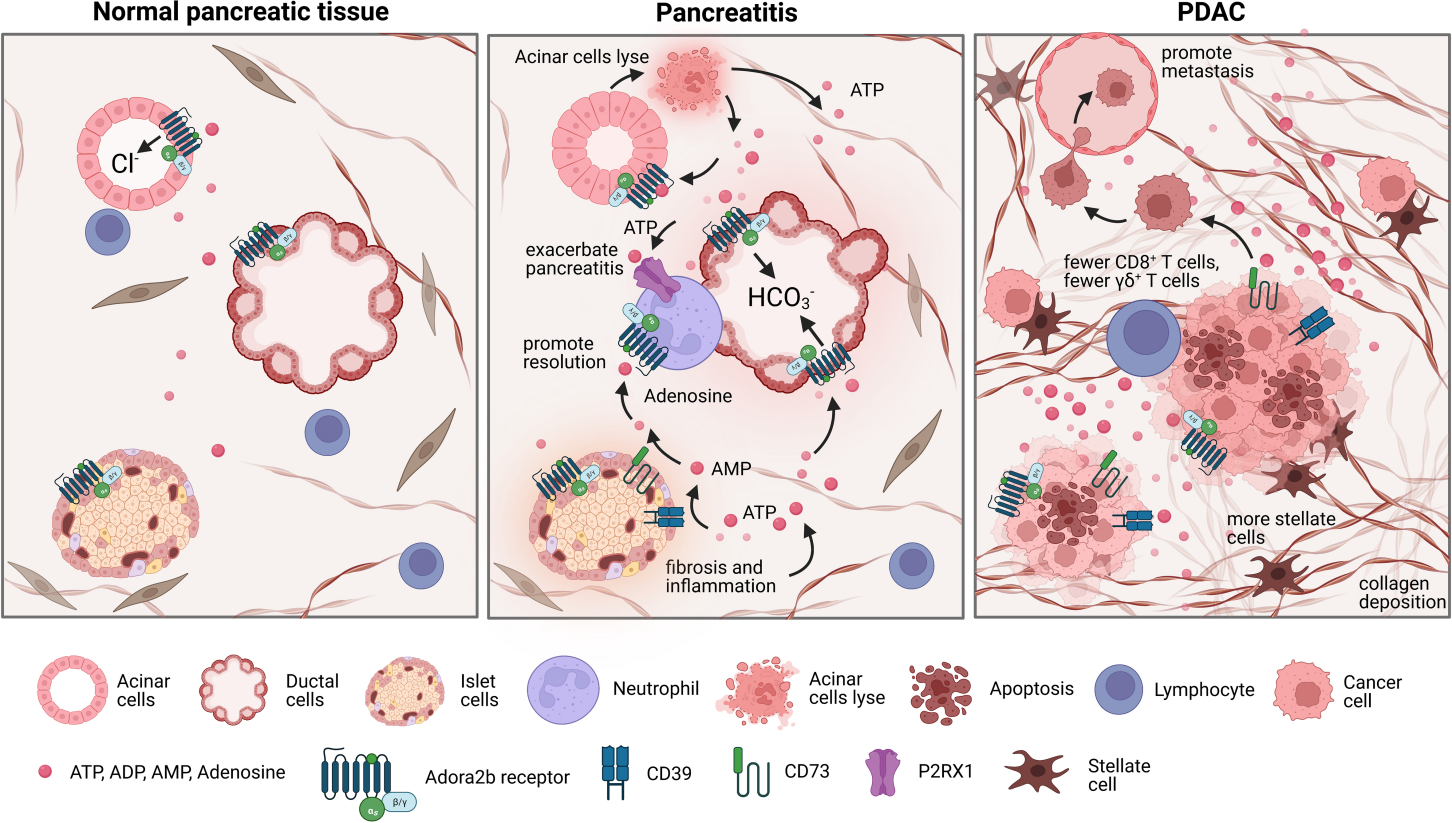
Changes in the pancreatic landscape in response to pancreatitis and pancreatic ductal adenocarcinoma (PDAC). Normal pancreas tissue is comprised of acinar cells that release digestive enzymes and Cl^-^ rich fluid, ductal cells that release bicarbonate pancreatic juice, and islet cells that maintain glucose homeostasis. However, during pancreatitis acinar cells lyse, releasing ATP into the extracellular environment, promoting elevated purinergic signaling which leads to altered bicarbonate secretion levels and exacerbates inflammation. In chronic pancreatitis, damage to islet cells contributes to increased fibrosis and inflammation, promoting high extracellular ATP levels and increased adenosine signaling. Neutrophils also contribute to exacerbating pancreatitis by expressing P2RX1 which promotes glycolytic metabolism. Contrarily, adenosine can inhibit the inflammatory function of neutrophils through Adora2b mediated deactivation, which partially promotes the resolution of pancreatitis. PDAC is characteristically immunosuppressive and possesses a dense desmoplastic stroma with a hypoxic necrotic core. In the necrotic core, there are high levels of extracellular ATP and higher levels of CD39 and CD73, which leads to an accumulation of adenosine in the TME which can then bind to Adora2b and contribute to immunosuppression. This also leads to fewer γδ+ T cells, more collagen deposition, and more stellate cells.

The pancreas is comprised of both endocrine and exocrine cells. Specifically related to exocrine function, acinar cells organize into acini and constitute 70-90% of pancreatic cells while 5-25% of exocrine pancreatic cells are ducts. Acinar cells are responsible for releasing digestive enzymes and Cl^-^ rich fluid, while ducts release bicarbonate pancreatic juice to neutralize stomach acidity and deliver acinar cell-derived enzymes to the duodenum (89, 90). The characteristic zymogen granules in acini store intracellular ATP at 10uM concentrations (91, 92). In a healthy pancreas, ATP is secreted by acinar cells into the ducts where P2 receptors regulate Cl^-^ and K^+^ ion channels, cAMP signaling, and transporters resulting in ductal secretion of NaHCO_3_-rich fluid (93). Acini and ducts have both been shown to express CD39 and CD73 which generate luminal adenosine that signals through ductal P1 receptors Adora2a and Adora2b which stimulate the cystic fibrosis membrane conductance regulator Cl^-^ channels important for ductal function (94). While less numerous, accounting for approximately 3-5% of pancreatic parenchyma, endocrine-functioning islet cells are critical for glucose homeostasis, and pancreatogenic (Type3c) diabetes can occur in a subset of patients with acute or recurrent acute pancreatitis (48, 95, 96). Both human and rodent ducts express adenosine receptors, with Adora2a and Adora2b being the most prevalent in these cells. When these receptors are stimulated, Cl^-^ channels are opened and allow ductal secretions to occur indicating purinergic signaling is important for pancreas function and homeostasis (29, 48, 97, 98) (**Figure 4**, left panel).

In the pathophysiology of acute pancreatitis, the enzymes zymogen and trypsinogen are released due to premature activation of acinar cells resulting in local parenchymal destruction and activation of inflammatory pathways. When acinar cells prematurely secrete enzymes, they also secrete ATP and other inflammatory signals into the extracellular environment (99–101). High extracellular ATP levels promote elevated purinergic signaling which leads to altered bicarbonate secretion from pancreatic ducts, ductal dilation, infiltration of innate immune cells, and increased severity of pancreatitis (11). Purinergic receptors are expressed on neutrophils and are key chemoattracts for these cells, which elevate pancreatic inflammation and the severity of pancreatitis. Thus, the conversion of ATP by CD39 and CD73 to adenosine is an important anti-inflammatory mechanism to return the pancreas to normal homeostasis after acute injury (102). Recent studies using single-cell RNA sequencing reveal CD73 is expressed in T cells and ductal cells in murine and human models of chronic pancreatitis (103). During chronic pancreatitis, not only are acinar cells severely injured, but also islet cells, with increased fibrosis and inflammation. This causes an extreme accumulation of extracellular ATP and exacerbated purinergic signaling (11) as well as increased infiltration of P2RX1 expressing neutrophils (102). Anti-inflammatory adenosine Adora2b signaling on ducts, neutrophils, and insulin-producing beta cells is therefore critical to promote healing after acute and chronic pancreatic injury (**Figure 4**, middle panel). Adora2b signaling reduces netosis formation and reduces oxidative burst from neutrophils, critical functions that reduce neutrophil-mediated inflammation during pancreatitis (104, 105). Future studies to determine the exact role of Adora2b receptor signaling in acute and chronic pancreatitis are important for future therapeutic considerations.

PDAC has a characteristically immunosuppressive TME where tumor cells coexist with exhausted and deactivated immune cells within a dense hypoxic desmoplastic stroma and necrotic tumor core (27). Understanding and targeting mechanistic triggers of immune suppression is one therapeutic approach being testing in preclinical and clinical trials. In a recent immunohistochemical study on human PDAC tissues, Jacoberger-Foissac et al. found that worse prognosis occurred only when patients present with elevated expression of both CD39 and CD73. When CD39 levels are high but CD73 levels are low, there is an increase of CD8^+^ T cells; however, this effect is not present when CD73 levels are also high, reaffirming that production of adenosine limits CD8^+^ T cell infiltration into PDAC tumors (6). Elevated expression of CD39 and CD73 has also been associated with fewer γδ+ T cells, more collagen deposition, and more proliferation of stellate cells indicating adenosine signaling may also be a critical determinant of fibrosis and desmoplasia in pancreatitis and pancreatic cancer (85, 106, 107). Three recent publications have utilized preclinical mouse models to evaluate the role of adenosine signaling in pancreatic cancer and have collectively shown genetic deletion of CD73 or treatment with CD73 small molecule inhibitors in syngeneic or genetic mouse models significantly reduces the development and progression of pancreatic cancer and promotes increased anti-tumor immunity; however, there are some differences in the models and findings which we want to highlight (4–6). In a publication by King et al, the authors performed a metabolic screen and found elevated CD73 correlated with aggressiveness of disease. The authors genetically deleted Nt5e/CD73 in murine PDAC cells and used an orthotopic model to show deletion of CD73 significantly ablated tumor growth and reduced the abundance of infiltrating MDSCs. They further show the anti-tumor immune response in Nt5e depleted tumors was associated with CD4^+^ and CD8^+^ T cells expressing IFN γ and showed the response was dependent on CD4^+^ T cells, but not CD8^+^ T cells (5). In a second publication by Jacoberger-Foissac et al, CD39 expression on CD8^+^ T cells was shown to suppress IFN γ production by T cells and transplantation of murine KPC tumors, myeloid expression of CD39 and CD73 and tumor expression of CD73 promoted polarization of myeloid cells to an M2 phenotype, which promoted PDAC growth and targeting both CD73 and CD39 significantly enhanced the anti-tumor T cell response. These findings were both done in the transplanted or orthotopic setting. Similarly, in the publication by Faraoni et al, inhibition of CD73 in murine genetic (spontaneous) models of pancreatic cancer, significantly reduced cancer development in spontaneous models with higher expression of CD73 in the neoplastic and cancer cells. Notably, pharmacologic inhibition of CD73 correlated with a significant increase in activated CD8+GZM+ T cells and F4/80+ cells in both genetic models. The authors then expanded these studies to a subcutaneous model to show inhibition of CD73 or the Adora2b receptor reduced the growth rate of murine KPC tumors. A limitation of the subcutaneous model is it does not recapitulate the microenvironment of the pancreas or the desmoplastic response in the pancreas. However, in this model, Faraoni et al. show the reduction in tumor growth using a small molecule inhibitor of Adora2b is dependent on CD8^+^ T cells. These studies were conducted to expand beyond the findings using CD73 inhibitors in spontaneous, orthotopic and subcutaneous models as we show in the publication by Faraoni et al, that PDAC patients with high *ADORA2b* have reduced survival and poor prognosis. In addition, we have shown using Quantiseq and The Cancer Immune Atlas analysis that patients with high *ADORA2b* or high CD73 have decreased NK cells, CD8^+^ T cells, B cells, and M2 macrophages (4). In studies using implantation of murine KPC tumors into WT or *Adora2b^-/-^* mice, we show a significant reduction in tumor growth in tumors arising in *Adora2b^-/-^* mice compared to WT mice. Pharmacologic inhibition of Adora2b also restrained tumor growth *in vivo*; however, the effect of the small molecule inhibitor was not present in tumor growth in CD8KO mice indicating adenosine signaling through Adora2b significantly restrains CD8^+^ T cell anti-tumor activity in PDAC (4) (**Figure 4**, right panel). These data indicate that co-inhibition of CD73 and Adora2b may provide additional therapeutic targeting to activate anti-tumor immunity and improve outcomes for PDAC patients.

## Adora2b function in metastasis

Greater than 90% of cancer-related deaths are due to metastasis, illustrating an urgent need for an improved understanding of mechanisms driving metastasis and ways to prevent metastases from forming. Traveling through the bloodstream, rogue cancer cells create metastatic cancer nodules that are highly resistant to therapies (108). In experimental mouse models of melanoma and triple-negative breast cancer metastasis, the incidence of metastasis is significantly decreased when mice are treated with an Adora2b antagonist (109). Similarly, genetic deletion of the Adora2b receptor in mouse and human triple-negative breast cancer cells reduces their metastatic capability *in vivo* (109), suggesting an important role for Adora2b in cancer metastasis. Recently, it was also shown that antagonizing Adora2b expression in gastric cancer cells increased the efficacy of cisplatin treatment (110). However, despite these promising results in melanoma, breast cancer, and gastric cancer cells, the specific role of Adora2b in metastatic development remains unknown. Metastasis is especially common in PDAC patients, due to the unfortunate ability of PDAC tumor cells to evade the exhausted and suppressed immune system. Future studies will be needed to further demonstrate the potential role of Adora2b in pancreatic cancer metastasis as well as their potential impact on this and other diseases.

## Experimental considerations for targeting autocrine and paracrine Adora2b signaling

### PDAC organoids and cell lines

Organoid models are a highly translational model system and provide an *ex vivo* approach to studying healthy pancreas and PDAC. Derived most from human or murine tissues, they are 3D and capable of self-renewal as well as spontaneous self-organization, providing a unique opportunity to study therapeutic approaches to augment personalized medicine, therapeutics, and mechanisms of resistance (111–114). Pancreatic organoids can also be orthotopically implanted after cryopreservation or genetic manipulation allowing more rapid studies of mechanistic drivers of PDAC development and metastasis *in vivo.* Noteworthy, it is important to mention that although organoids offer an interesting platform to test therapeutic drugs and can be applied to many different cell types and diseases, they still lack a high-fidelity cell type composition, have limited maturation, and have an atypical physiology which does not always can recapitulate or mimic interactions between molecules when compared to the physiologically normal and/or tumor microenvironments, which limit their applicability and reliability for certain tumor studies (115). If organoid models are not available, human PDAC cell lines can also be used as an *in vitro* mechanistic approach to study cell autonomous and non-cell autonomous purinergic signaling. Established cell lines from human PDAC primary tumors are BxPC-3, Capan-2, HPAC, MIA PaCa-2, and Panc-1. BxPC-3 is the only cell line mentioned which is wild type for *KRAS* and does not represent the majority of PDAC tumors, which have somatic mutations in *KRAS* (116). For each of these human cell lines, experiments can be done with Adora2b agonists, Adora2b antagonists, siRNA, or CRISPR/Cas9 mediated genetic deletions, to study the cell-autonomous upstream and downstream effects of adenosine signaling through the Adora2b receptor. The KPC cell line is also a very common murine PDAC cell line with mutations in *Trp53* and *Kras.*

### Mouse models

Mouse models are essential to studying pancreatic cancer and there are numerous models which would be useful to study the Adora2b receptor and its role in PDAC. First, there are syngeneic models utilizing subcutaneous or orthotopic implantation of KPC cells into the flank, pancreas, spleen, or any combination of these injection sites. These models are useful for studying treatment options using Adora2b antagonist compounds in primary tumors and metastatic sites (4). There are also genetically engineered mouse (GEM) models that can be used, such as the KPC and *Pdx : Cre;LsL-Kras^G12D^* (KC) models. The KPC mice have mutations in *Kras, mutations or* genetic deletion of *Trp53*, and use Cre-Lox technology through Cre recombinase gene insertion into *Pdx-1* or *Ptf1a* (p48-Cre) coding exons. KPC mice begin to develop PDAC precursor lesions around 8-10 weeks of age and have PDAC by 4 months of age (117). KC mice are advantageous for prevention studies as they have slow development from PanIN to PDAC over a time frame of 12-15 months (118). Future studies in GEM models could also be used to test different Adora2b antagonist compounds *in vivo* and to study immune cell interactions in the preventive or therapeutic setting. Using cell-specific inducible CreER alleles crossed to an Adora2b floxed allele, genetically engineered mouse GEM models can be generated with genetic deletion of *Adora2b* in specific cells or tissues. Mice without Adora2b receptors in the defined immune cells, stromal cells, or vasculature could also be useful to study the role of the receptor in PDAC in the future.

#### Adora2b agonist and antagonist compounds

**Figure 5:**
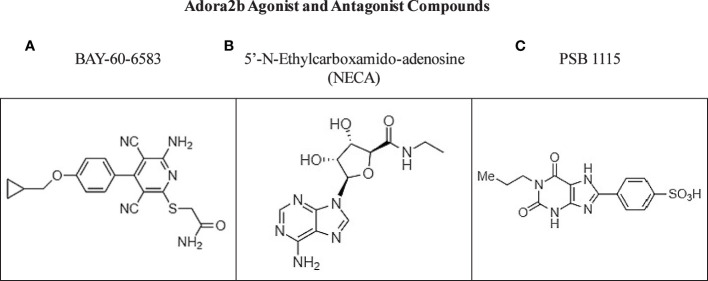
Commercially available compounds targeting Adora2b. **(A, B)** Chemical structures of Adora2b agonist compounds BAY-60-6583 and NECA. **(C)** Chemical structure of Adora2b antagonist compound PSB 1115.

Selective adenosine agonists and antagonists have been described for the Adora2b receptor and support the protective and anti-inflammatory mechanistic consequences of Adora2b signaling. Particularly in pancreatic diseases, 5’-N-ethylcarboxamidoadenosine, commonly abbreviated as NECA, was recently administered in a model of pancreatitis and described as a suitable Adora2b agonist which may be involved in tissue regeneration and restraint of MPO accumulation and metaplasia during acute pancreatitis; however, no specific therapeutic applications of NECA have been described to date in the clinic (29). Though studies have shown short-term adenosine exposure is highly effective at reducing pain and inflammation, high levels of adenosine have been reported to increase tissue damage and may increase inflammation and potentiate protumor adenosine signaling (119). For these reasons, Adora2b antagonist compounds could be potential therapies in cancer (120). Notably, some of the Adora2b antagonists have been described to decrease the secretory rate of the pancreas by 25% and increase insulin production levels (48). Mice bearing KPC subcutaneous tumors treated with Adora2b antagonist PSB1115, presented with significantly decreased KPC tumor growth and significantly decreased fibrosis measured by IHC for α-SMA. These studies highlight the complex dynamics of this pathway and the urgent need for preclinical and clinical evaluation of targeting Adora2b receptor signaling to better deduce its role in immunity, fibrosis, and cancer (4) (**Figure 5**).

### Current therapeutic opportunities and clinical trials

Studies have shown there is an estimated time of 10 years between the moment at which a pancreatic epithelial cell undergoes an oncogenic hit and the time of diagnosis (121), which provides a wide window of opportunity for the detection and prevention of precancerous lesions including pancreatic intraepithelial neoplasia (PanIN). A recent study of healthy human pancreata has shown PanIN are present in individuals irrespective of age and these PanIN have transcriptional signatures that share similarities to cancer cells (122). Despite this recent finding, the lack of technology or systemic biomarkers available for performing early detection allows precursor lesions to progress to a point where, when detected, PDAC is diagnosed at advanced stages and is unresectable in 70-80% of patients diagnosed. Thus, there is a need to test and evaluate new approaches in patients with locally advanced or borderline resectable pancreatic cancer including the use of immunomodulators in the neoadjuvant setting. Considering recent publications showing Adora2b reduces the cytotoxic functionality of NKT and CD8^+^ T cells, there is an increased premise to evaluate inhibiting Adora2b signaling in the prevention setting. Targeting the adenosine signaling pathway at the preclinical stage has been an intense area of study in recent years and future studies in GEM models of PanIN initiation and progression to PDAC would aid in determining if targeting this pathway has clinical promise. Preclinical studies utilizing checkpoint blockade combined with ectoenzyme blockade approach through inhibition of CD39, CD73, PD-1/PDL-1, and the various adenosine receptors may show enhanced antitumor immunity, decreased tumor initiation, and metastasis, but have not yet been evaluated. As a tightly balanced extracellular amount of both adenosine and ATP is needed to maintain an adequate immune response, therapeutic combinations of CD39 with PD1/PDL1 with and without chemotherapy are being studied (123, 124). There are also ongoing clinical trials targeting the Adora2a receptor in combination with CD73 or PDL-1 inhibitors (**Table 1**) (125–128). Adora2a blockade studies are also ongoing in combination with PD-1, PDL-1, or chemotherapy (11). However, there are no current clinical trials specifically targeting the Adora2b receptor. It is important to consider the complex interactions between purinergic receptors and ATP/ADP/adenosine signaling, because receptor blockade may impact unwanted cell types and promote unintended effects on other receptors (11). For clinical and therapeutic considerations, there is also a need to evaluate the role of the Adora2b receptor in regulating perineural infiltration, fibrosis, and vasculature as the PDAC microenvironment is dynamic and recent studies have shown multiple subtypes of PDAC can co-exist in patients with pancreatic cancer.

**Table 1:** Current clinical trials.

Target	Drug +/- combination therapy	Tumor	Identifier	Study Phase
Adora2a	Ciforadenant (A2A inhibitor) + atezolizumab (PD-L1 inhibitor)	Incurable Cancers	NCT02655822	Phase I/Ib
Adora2a	NIR178 (A2A inhibitor) + PDR001 (anti-PD-1 mAb)	Solid tumors and Non- Hodgkin Lymphoma	NCT03207867	Phase II
CD73 +/- Adora2a	CPI-006 (anti-CD73 mAb) +/- ciforadenant (A2A inhibitor) +/- pembrolizumab (anti-PD1 mAb)	Solid tumors, including PDAC	NCT03454451	Phase I/Ib
CD73 +/- Adora2a	NZV930 (anti-CD73 mAb) +/- PDR001 (anti-PD-1 mAb) +/- NIR178 (A2A inhibitor)	Solid tumors, including PDAC	NCT03549000	Phase I/Ib

Current ongoing clinical trials targeting adenosine receptors for treatment in pancreatic cancer and other tumors.

## Challenges to the field

Pancreatic cancer is a particularly challenging field to study, as it is extremely complex, and tumor genetic and histologic heterogeneity is prominent when comparing patient tumor samples. The advent of sequencing human PDAC tumors has revealed PDAC subtypes (129–132) and Squamous and Basal subtypes have been reported to have the highest expression of CD73 (4) indicating they may have more pronounced intratumoral levels of adenosine. One of the challenges to this field is that most studies of adenosine receptor signaling, and interactions are performed in mouse models, which may not translate directly into humans. This limitation, while applicable to most, if not all preclinical studies, makes it difficult to accurately translate therapies targeting adenosine receptors into human patients, as there may be unintended side effects or limitations of small molecule inhibitor activity or delivery not observed in murine models. Another complication to using mouse models is the immense time requirement to breed genetically engineered mice that more accurately represent human PDAC progression. Despite these limitations, more preclinical and clinical studies need to be done to more accurately evaluate the role of adenosine signaling and possible resistance mechanisms to small molecular inhibitors targeting this pathway in cancer as most studies conducted on extracellular purinergic and adenosine signaling have been in diseases other than pancreatic cancer including acute lung disease, acute liver disease, asthma, diabetes, myocardial ischemia, sickle cell disease, and IBD. Another challenge related to the field of use of Adora2b small molecule inhibitors for immunotherapeutic consideration is that few studies have been performed exploring specifically the Adora2b receptor on individual tumor cells, fibroblasts, or immune cell types in the context of the tumor microenvironment. Studies using human or murine organoid cultures and genetic deletion of Adora2b or pharmacologic inhibition will aid in scientific understanding of the mechanistic consequences of Adora2b expression in pancreatic cancer and also help determine if different PDAC subtypes respond differently to Adora2b inhibition. In addition, the role of the gut microbiome or intrapancreatic bacteria or fungi may also elevate adenosine or inosine levels elevating the importance of targeting this pathway for cancer treatment (133, 134). Future studies evaluating the functional consequences of Adora2b receptor signaling in different innate and adaptive immune cell types and interactions are also desperately needed to advance immunotherapies in this field.

## Discussion

Pancreatic ductal adenocarcinoma is aggressive, resistant to therapy, and successful treatments are desperately needed, as current options have not yet resulted in significant changes in overall survival. In this review, we discuss literature related to the function of Adora2b, a low-affinity adenosine receptor prominently known for its role in reducing inflammation. The hypoxic TME of PDAC creates a unique niche where CD73, CD39, and Adora2b are elevated resulting in dynamic changes in concentrations of ATP and extracellular adenosine. The ENT1 transporter promotes sensitivity to chemotherapy in PDAC patients and high expression has strong prognostic implications for improved outcomes in PDAC (135). ENT1 is critical for regulating nucleoside concentrations and under hypoxic conditions regulates adenosine receptor signaling (136) indicating another possible combination therapeutic approach, as ENT1 is important for the transport of nucleotides into and out of the cell. Future studies deducing the entire pathway in cancer development and metastasis will aid in determining the utility of targeting this pathway to improve patient outcomes.

Another important consideration is the four P1 adenosine receptors have divergent roles dependent on cell type expression and concentrations of ligands. Of the four receptors, Adora2a and Adora2b have been reported as high in PDAC and are overexpressed in the pancreas during pancreatic cancer; yet only high expression of Adora2b receptor was shown to correlate with significantly reduced survival in PDAC patients. We recently published that patients with high ADORA2B have reduced CD8^+^ T cells and NK cells indicating inhibiting this receptor may have utility in recruiting activated CD8^+^ T cells and NKT cells to target PDAC (4). However, these efforts are complicated by the fact that Adora2b is present on virtually all myeloid and lymphoid lineage cells, and activation of the receptor on these cells can alter their functionality and contribute to dynamic changes in immune cell function in the TME. A critical consideration for future trials is understanding patient-specific levels of CD73, Adora2b and ATP, ADP, and adenosine available to signal through P2 or P1 receptors. Adenosine is rapidly taken back into cells and converted to inosine by ADA, which has also been shown to have immunosuppressive consequences in cancer models (133). Thus, understanding the full context of this incredibly complex signaling pathway including Adora2b functionality warrants further consideration and research efforts. Clinical trials where patient samples are available pre and post-treatment are urgently needed to determine if targeting this pathway will improve overall survival. Trials in both the neoadjuvant and adjuvant setting should be conducted due to recent publications showing the Adora2 receptors can promote tumor growth, metastasis and reduce CD8^+^ T cell anti-tumor immunity predominantly in preclinical models (3, 4, 11, 120, 123–126, 137–144).

## Author contributions

LS wrote the original draft. EF, XY, WR, HE, and JB-L edited the manuscript. WR made the Figures and LS made the Table. All authors contributed to the article and approved the submitted version.
